# Introduction to “Geoffrey Bodenhausen Festschrift”

**DOI:** 10.5194/mr-4-111-2023

**Published:** 2023-05-02

**Authors:** Daniel Abergel, Fabien Ferrage

**Affiliations:** Département de Chimie, LBM, UMR 7203, École Normale Supérieure, PSL University, Paris, France

## Graphical abstract


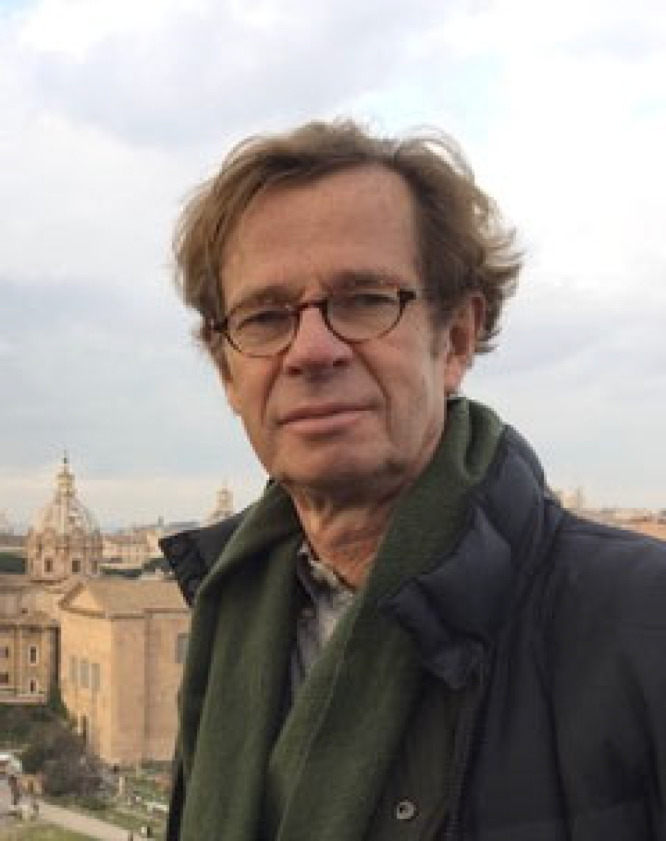


This special issue of *Magnetic Resonance* is dedicated to Geoffrey Bodenhausen on the occasion of his 70th birthday. For more than 4 decades, his name has been associated with some of the major developments covering many domains of NMR spectroscopy.

Born on 7 May 1951 in The Hague in the Netherlands, Geoffrey Bodenhausen was educated at the École Internationale de Genève. Later, after graduating from ETH Zürich, he started his life as a scientific globetrotter, exploring a large variety of exciting areas in NMR and the planet. This started at the University of Oxford, where he undertook his NMR career under the auspices and guidance of Ray Freeman, in the early days of 2D NMR. There, he contributed to the development of correlation spectroscopy and selective excitation, some aspects that he has consistently explored since.

Leaving the Old Continent for the New World, Geoffrey contributed to the development of such domains as multiple-quantum spectroscopy, relaxation, and phase cycling, which are all central to modern NMR spectroscopy. Not to mention the invention of the emblematic heteronuclear single-quantum coherence spectroscopy (HSQC) experiment, which is the model of heteronuclear multi-pulse multidimensional NMR.

Bodenhausen's early excursions into the domains of liquid crystals with Robert and Regitze Vold and solid-state NMR with Bob Griffin laid the foundations for later methodological developments.

Back at ETH Zürich in 1980, in the research group of Richard Ernst – a laboratory that shaped NMR spectroscopy for decades – he continued to work on diverse aspects of NMR, including 2D spectroscopy, relaxation, and solid-state NMR and contributed to the systematic quantum-mechanical description of NMR in the form of coherence transfer pathways, phase cycles, product-operator formalism, and so on. This intense period also led to the celebrated book, *Principles of Nuclear Magnetic Resonance in One and Two Dimensions*, with Alexander Wokaun and Richard Ernst.

Settling on the shore of Lake Geneva (Lac Léman) in 1985, Bodenhausen freely developed his approach to NMR at Université de Lausanne (UNIL) and then at École Polytechnique Fédérale de Lausanne (EPFL). In parallel, he temporarily moved to the National High Magnetic Field Laboratory (NHMFL) in Tallahassee, Florida, USA, before building a new NMR laboratory at École Normale Supérieure (ENS) in Paris. For Bodenhausen, advancing methodology has been the opportunity to do an in-depth exploration of fundamental phenomena, and vice versa, while never forgetting spectroscopy's essential role in extracting chemical information. Spin relaxation is one such domain, where, in addition to earlier contributions, he developed numerous methods based on cross-correlated effects to shed a brighter light on the structural and dynamical properties of (bio)molecules.

Over the years, Bodenhausen continuously developed spin-engineering techniques, among which selective pulses, signal processing, and, more recently, long-lived states and dynamic nuclear polarization, while demonstrating his inextinguishable curiosity through works on exotic topics, such as radiation damping or ${}^{131}$ Xe spectroscopy for the study of surfaces, to name but a few. The breadth of Bodenhausen's interests is best illustrated by his many contributions not only to solution- and solid-state NMR but also in MRI and even mass spectrometry, with the introduction of the concept of 2D spectroscopy to Fourier transform ion cyclotron resonance.

Beyond pure scientific achievements, Bodenhausen has been actively serving the NMR community by taking part in the organization of international meetings, first at the Experimental NMR conference (ENC) and then at the European Magnetic Resonance congress (EUROMAR), as the founding chairperson of the board of trustees. His many editorial activities are not limited to the positions of editor of several NMR journals but, importantly, to promoting open-access to scientific literature, which led to the creation of this journal, *Magnetic Resonance*, which is backed by the Groupement AMPERE (Atomes et Molécules Par Études Radio-Électriques).

Last, but not least, Bodenhausen has trained dozens of young students that have spread out over the academic world and in industry. Offering them both great freedom of research and many opportunities, he nurtured several generations of scientists, many of whom have had successful careers. On a more personal note, sharing over 20 years of lab life has shown us an ever-enthusiastic scientist, who is eager to promote new ideas, always curious, demanding but supportive, and an intrepid lover of science.

This special issue, rich with 28 contributions, illustrates both the breadth of Geoffrey Bodenhausen's scientific interests and his influence in the NMR community. Contributions cover a range of diverse topics, including solid-state NMR, with the use of selective pulses to reduce radiofrequency inhomogeneity in solid-state magic-angle spinning (MAS) spectroscopy (Aebischer et al., 2020), methods for the optimization of polarization transfer between spin 1/2 and quadrupolar nuclei under MAS (Gómez et al., 2021), in addition to strategies for the elucidation of the structural properties of kidney stones (Leroy et al., 2021). A variety of contributions in solution-state biomolecular NMR is included in this special issue, with a new approach to the analysis of relaxation rates (Crawley and Palmer, 2021), improved isotope filters (Marincin et al., 2021), the investigation of free conformations in ligand binding (Balazs et al., 2021), methods for the selective detection of proline residues (Felli et al., 2021), the determination of backbone motions in disordered proteins (Kauffmann et al., 2021), investigations of fluoroprolines in disordered proteins (Sinnaeve et al., 2021), the use of accordion spectroscopy to measure transverse relaxation rates (Wernersson et al., 2021), and new proton-only methods to probe excited states in nucleic acids (Liu et al., 2021).

More solution-state NMR contributions focus on the analysis of scalar couplings with deconvolution methods (Jeannerat and Cobas, 2021), virtual decoupling in the context of metabolomics (Charlier et al., 2021), and selective methods (MacKinnon et al., 2021) and investigations of the sensitivity requirement (Guest et al., 2021) in diffusion NMR.

Slightly more exotic topics cover the exploration of scalar couplings in a field-cycling experiment (Zhukov et al., 2020), a review of the broad range of applications of the NMR-MOUSE^®^ (Blümich and Anders, 2021), the effect of gradient echoes on spin noise (Rodin et al., 2021), and, of course, long-lived states (Teleanu and Vasos, 2021), as well as a comparative investigation of approaches to relaxation theory (Rodin and Abergel, 2022).

Last, but not least, a series of original contributions present methods and theoretical advances in the field of hyperpolarized NMR, which has been the focus of Bodenhausen's work over the past 15 years, from dynamic nuclear polarization (DNP) at room temperature (Miyanishi et al., 2021), the detection of hyperpolarized ${}^{129}$ Xe at a low magnetic field (Chighine et al., 2021), technological advances for dissolution DNP (Kress et al., 2021; Elliott et al., 2021) and bullet DNP (Kouril et al., 2021), an analysis of enzyme kinetics in the context of dissolution DNP (Eykyn et al., 2021), theoretical explorations of the limits of Liouville space (Levitt and Bengs, 2021), and experimental investigations of quantum-rotor-induced polarization (Dietrich et al., 2021).

Editing this special issue was very stimulating, as we interacted with NMR scientists practicing many flavors of NMR, and we hope readers will be very interested in reading these many contributions.

Finally, we could not write this note without mentioning our late friend Konstantin Ivanov, who gave the impulse for building a special issue in honor of Geoffrey. Konstantin Ivanov and Geoffrey Bodenhausen had met a few years before, and since then, several of us in the laboratory had the chance to collaborate with him on multiple occasions. We discovered, beyond the sharp-minded, extremely talented scientist and congenial colleague, a genuine and generous friend, who sadly had an untimely passing during the COVID-19 pandemic.
